# Gasotransmitters in Gametogenesis and Early Development: Holy Trinity for Assisted Reproductive Technology—A Review

**DOI:** 10.1155/2016/1730750

**Published:** 2016-08-08

**Authors:** Jan Nevoral, Jean-Francois Bodart, Jaroslav Petr

**Affiliations:** ^1^Laboratory of Reproductive Medicine of Biomedical Center, Faculty of Medicine in Pilsen, Charles University in Prague, Alej Svobody 1655/76, 323 00 Pilsen, Czech Republic; ^2^Department of Histology and Embryology, Faculty of Medicine in Pilsen, Charles University in Prague, Alej Svobody 1655/76, 323 00 Pilsen, Czech Republic; ^3^Team Régulation des Signaux de Division, UMR 8576 CNRS, Université Lille 1, Sciences et Technologies, FR 3688 CNRS, 596 55 Villeneuve d'Ascq Cedex, France; ^4^Institute of Animal Science, Pratelstvi 815, 104 00 Praha Uhrineves, Czech Republic

## Abstract

Creation of both gametes, sperm and oocyte, and their fusion during fertilization are essential step for beginning of life. Although molecular mechanisms regulating gametogenesis, fertilization, and early embryonic development are still subjected to intensive study, a lot of phenomena remain unclear. Based on our best knowledge and own results, we consider gasotransmitters to be essential for various signalisation in oocytes and embryos. In accordance with nitric oxide (NO) and hydrogen sulfide (H_2_S) physiological necessity, their involvement during oocyte maturation and regulative role in fertilization followed by embryonic development have been described. During these processes, NO- and H_2_S-derived posttranslational modifications represent the main mode of their regulative effect. While NO represent the most understood gasotransmitter and H_2_S is still intensively studied gasotransmitter, appreciation of carbon monoxide (CO) role in reproduction is still missing. Overall understanding of gasotransmitters including their interaction is promising for reproductive medicine and assisted reproductive technologies (ART), because these approaches contend with failure of* in vitro* assisted reproduction.

## 1. Introduction

Human reproductive medicine and assisted reproductive technologies (ART) have been gaining increasing significance, dealing with human reproduction failure. Doubtlessly, the oocyte and sperm are crucial cells for assisted reproduction because these haploid gametes are required to build a diploid zygote, capable of further development. Female and male gametes exhibit different morphological features and, excluding brought genome, they differently contribute to embryo formation. While centrosomes, small noncoding RNAs, and posttranslationally modified residual histones are sperm-inherited, oocytes provide mitochondria, mRNAs (distributed according to a specific pattern), histones, metabolic enzymes, and cytoplasmic factors to sustain development, as summarized elsewhere [1–5]. Hence, one has to consider the oocyte as a microenvironment filled with a precisely balanced cocktail of the numerous factors that are essential for embryonic development. Also, oocytes offer physical environments favourable to self-organizing process like division spindle assembly. Upon fertilization, a succession of mitotic divisions is triggered; the transition from maternal to zygotic mRNAs transcription and transformation of a low organized cellular mass into a blastocyst will occur prior to implantation. Together with gametogenesis and DNA integrity maintenance, these events are of high interest for ART. Indeed, any failure in these processes will impact severely the embryo's fate. Untangling the processes at the molecular and cellular levels is crucial for ART and we should underline that the effects of many contributors, besides the main regulators of gametogenesis and early embryogenesis, remain uncovered.

Oocyte maturation, which can be simulated in* in vitro* conditions, deserves particular attention because meiotic division and achievement of developmental competence are finalized during this short and extremely important period (summarized in [5]). The quality of matured oocytes is decisive for the fertilization rate, as a result of sperm penetration and complex oocyte changes including cortical granule exocytosis-prevented polyspermy and oocyte activation for embryonic development [6–8]. In fact, the early embryonic development, where high-quality blastocyst is optimal for embryo transfer into the recipient body, is decisive for the success of ART [9, 10]. Numerous factors have been identified to play different roles in chromosome segregation and developmental competence achievement, regulating kinases, structural cytoskeletal proteins, enough histones, and second messengers (cAMP, cGMP, and Ca^2+^ ions) [11–14]. In addition to these known key factors, gaseous molecules with signal transduction ability, hence named gasotransmitters [15–17], have been involved in the oogenesis as well [18, 19]. Their impact is acknowledged along with a better understanding of gasotransmitters' signalling pathways. Moreover, recent observations point out imperfect* in vitro* imitation [20] and some gasotransmitter signalisation seems to be lacking in complete gametes' maturation and early embryogenesis.

Only matured oocytes are able to go through* in vitro* fertilization, a key technique of assisted reproduction [21]. Fertilization consists in the interactions of male and female gametes leading to embryonic development. The high cell division rate, typical of this period, is highly sensitive to well-orchestrated cell cycle regulation [2, 22, 23]. Oocyte maturation and early embryonic development persist as delicate steps for* in vitro* approaches, calling for ART improvement. Nevertheless, gasotransmitters rise expectations due to their broad physiological effect and promising results of gasotransmitters supplementation.

The aim of this review is to compare the biological necessity of all three gasotransmitters in the oocyte and embryo, observing their* in vitro* culture in ART, as a key factor for creating a new individual. This comparison highlights protein posttranslational modifications as crucial molecular action of gasotransmitters during oogenesis and preimplantation embryonic development.

## 2. Gasotransmission in Female Reproductive Processes 

### 2.1. NO as a “Yes Signal” for Fertilization and Early Development

**Figure 1:**
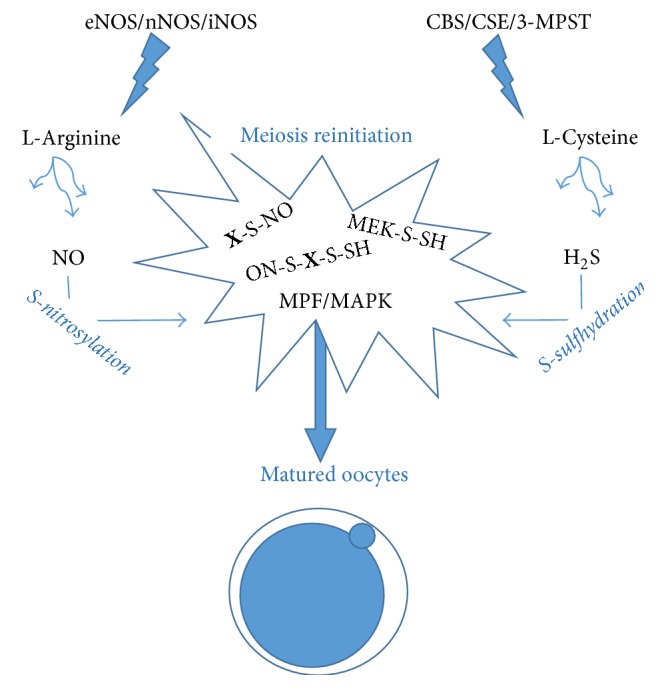
Gasotransmission in oocytes, resulting in S-sulfhydration and nitrosylation of various factors. Both gasotransmitters NO and H_2_S are enzymatically released, respectively, from L-arginine and L-cysteine. Subsequently, NO- and/or H_2_S-posttranslationally modified proteins lead to MPF/MAPK-orchestrated meiotic maturation reinitiation (equal to GVBD, germinal vesicle breakdown) and completion (with extruded polar body and small particles visible in perivitelline space). S-sulfhydration of MEK, upstream MAPK kinase, is known [61] and more S-sulfhydrated factors are considered. In addition to S-sulfhydration, S-nitrosylation seems to be exclusive mechanism of NO-regulated oocyte maturation [34]. Disclosure of complete “S-sulfhydration” and “S-nitrosylation” is still lacking (**X**-S-SH,** X**-S-NO) and we can assume wide protein index underwent this posttranslational modifications as well as NO-H_2_S intraprotein cross-talking (HS-S-**X**-S-NO).

Only matured gametes, which underwent adequate changes, are capable of fertilization. These changes involve especially oocyte maturation, sperm capacitation, and acrosome reaction and are an essential prerequisite for both successful fertilization and further embryonic development. Biochemical changes regulate gametes' changes and their interactions during fertilization process. Originally, these changes were believed to be exclusively regulated* via* kinase signalling, such as protein kinase A- (PKA-) M-phase/maturation promoting factor- (MPF) mitogen-activated protein kinase (MAPK) and calmodulin-dependent protein kinase II (CaMKII), either directly dependent or indirectly regulated by molecules of second messengers, Ca^2+^ and cAMP. In addition to these two messengers, the involvement of NO, a small gaseous molecule, in cell signalling of physiological processes has been described [24, 25]. Along with NO, gasotransmitters H_2_S and CO were suggested to participate in the above-mentioned processes as well [17, 26–28] (see Figure 1).

NO represents the most read-up gasotransmitter, with ability to regulate molecular processes in gametes and embryo [29–31]. All NO synthases, that is, endothelial (eNOS), neuronal (nNOS), and inducible (iNOS), are present in mammalian oocyte with various subcellular localization, where they are essential for endogenous production of NO and its cell signalisation [32, 33]. NO action leads to ovulation of matured and fertilizable oocytes [31, 32] as a result of reinitiation of oocyte meiosis and correct oocyte maturation [18, 31, 32]. Accordingly, NO level in oocytes of young mice is significantly higher than old animals and NO antiaging effect is obvious [34]. On the contrary, increased eNOS expression accompanies improved mouse oocyte quality after estrogen administration [35]. One of NO action modes, S-nitrosylation of proteins, has been observed in oocytes during meiotic maturation [36]. However, NO is able to stimulate soluble guanylate cyclase (sGC), which is a NO-specific receptor, in cGMP production and thus NO increases protein kinase G (PKG) activity [37–40]. On the other hand, S-nitrosylation of sGC affects the decreasing responsiveness to NO in somatic cells and molecular mechanism-dependent dual effect of NO is obvious [41]. In contrast to oocyte maturation [38], NO-sGC-cGMP-PKG signal pathway is capable of inducing spontaneous oocyte activation and subsequent parthenogenetic development [42]. NO-induced oocyte activation indicates a pulsation pattern of NO action in porcine oocytes [43]. Based on an observation of* Xenopus* oocytes, the parthenogenetic NO effect is Ca^2+^-dependent and occurs due to MAPK inactivation [44].

**Figure 2:**
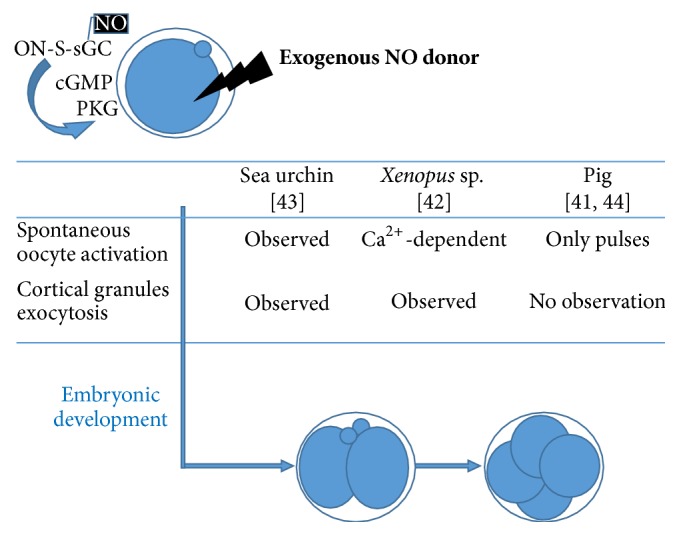
NO action in oocyte activation is evolutionary inconsistent. The NO/sCG/cGMP/PKG signal pathways are presumed, where dual NO effect on sCG, resulting in its S-nitrosylation and NO binding, is expectable. Obviously, dependency of fertilization and oocyte activation, followed by cleavage and the second polar bod extrusion, is shaded in evolutionary more developed organisms, where fulfilment of certain conditions (Ca^2+^ presence, pulsative character of NO) is necessary.

While NO might promote but is dispensable for* Xenopus* and mammals oocyte activation, NO is essential for oocyte activation event during the fertilization process in sea urchin oocytes [45]. In accordance with this variable effect, cortical granules exocytosis has been reported in* Xenopus* oocytes [44] but not in porcine oocytes [46]. The interspecies differences of NO action during fertilization are obvious and NO seems to be even nonessential during mammalian fertilization (Figure 2). The ambiguous NO necessity could be a result of a more diverse NO effect when NO is associated with inflammation and/or oxidative stress [47, 48]. Accordingly, the role of NO during subsequent embryonic development after fertilization remains controversial [49–51] for inflammation (endometriosis), accompanying NO [52, 53] and protein nitration [54]. However, creation of secondary products of NO interactions seems to be one of possible mechanisms of NO negative action [55]. The physiological role of NO in embryogenesis is still unexceptionable when NO is involvement in embryonic stem cell differentiation through transcriptional factors [56]. Therefore, NO is able to be considered as trigger for oocyte maturation and fertilization as well as subsequent embryonic development.

### 2.2. H_2_S in Gametogenesis and Embryo Development

S-sulfhydration, another gasotransmitter-derived posttranslational modification, is supposed to be a prime way of H_2_S molecular action [57, 58] without known H_2_S-specific receptors. In contrast to NO, little is known about H_2_S and S-sulfhydration involvement in gametogenesis and embryonic development. Nevertheless, all three H_2_S-releasing enzymes, CBS, CSE, and 3-MPST, were observed in porcine oocyte and surrounding cumulus cells [59]. This observation is in accordance with earlier finding of H_2_S involvement in folliculogenesis and oocyte maturation [19, 20]. The necessity of H_2_S in matured oocytes interferes with the contribution to developmental competence acquirement and subsequent embryonic development [20]. In addition, there is the observation of a protective effect of H_2_S against oocyte aging and H_2_S-positively affected further embryonic development [60]. Physiological action of endogenously released H_2_S immediately in oocyte has been described and modified kinase activity of MPF and MAPK has been observed [20, 59, 60]. S-sulfhydration of these kinases and their upregulated factors are presumable. Activating S-sulfhydration of MEK, leading to MAPK signalling [61], confirms this assumption and the findings mean that S-sulfhydration is crucial for enzyme activity and shift its significance to protein phosphorylation.

However, in contrast to the essential and protective effect of H_2_S in mammalian oocytes, our own observation of oxidative stress-like effect of H_2_S in* Xenopus* oocytes indicates less conservative evolutionary mechanism through species. Moreover, some findings support that H_2_S action is at least comparable to reactive oxygen species (ROS) throughout reactive sulfide species (RSS) creation [62–64].

Although the role of the third gasotransmitter, CO, remains uncovered, the necessity of gasotransmitters for male and female reproduction including fertilization and embryonic development is unquestionable. Accordingly, S-nitrosylation and sulfhydration of sulphur amino acid cysteine seem to be crucial protein posttranslational modifications for reproductive processes and their understanding brings relevant possibilities for ART.

## 3. An Increasing Attractiveness of S-Nitrosylation and S-Sulfhydration

Decades of research have established a high potential for NO and S-nitrosylation in controlling cellular mechanisms. Indeed, both NO and H_2_S might engage in protein short-lived covalent reactions, which modulate proteins structure and functions. NO builds its signalling activity by binding to sulfhydryl groups of cysteine residues in target proteins. The latter process is called S-nitrosylation. In a similar manner, S-sulfhydration is a posttranslational modification of specific residues, through the formation of persulfide (-SSH) bonds. Both sulfhydration and S-nitrosylation are reversible.

There is a broad spectrum of S-nitrosylated proteins. An exhaustive list would be beyond the scope of this review. Nevertheless, it is to note that nitrosylated proteins include cytoskeleton, cell migration, cell cycle, and antiapoptotic proteins, as well as proteins involved in transcription and protein synthesis [65–69]. In a similar way, protein-SSH formation is now admitted to mediate in a fundamental manner the cellular signalling by H_2_S, based on the detection of S-sulfhydrated proteins and on the demonstration of their perturbed functions [70]. Spatial environments of the modified residues drive the impact of S-sulfhydration on protein function. For example, it may protect residues from oxidation under oxidative stress and therefore may sustain protein activities.

### 3.1. From Cell Cycle to Implantation, Potential Roles for S-Nitrosylation

Therefore, S-nitrosylation is a well-established posttranslation modification, whose potential involvements at physiological level in oocytes and embryos go from cell cycle regulation (meiotic transition, segmentation) to embryo survival and implantation.

Indeed, S-nitrosylation targets can be found within main modulators of meiosis progression or cell cycle progression and their regulators. Though the M-phase promoting factor, made up with cyclin B and cyclin-dependent kinase 1 (CDK1), was not reported to be itself S-nitrosylated, the S-nitrosylation of CDKs was observed for CDK2, CDK5, and CDK6 [71–73]. While CDK2-nitrosylation increases its activity independently of any effects on protein levels expression, the effect of S-nitrosylation on CDK5 and CDK6 remains elusive. S-nitrosylation of cyclin B was sought in HL-60 cells, but not observed [72]. No S-nitrosylation was reported for polo-like kinases (PLKs), anaphase promoting factor/cyclosome (APC/C), WEE1, and MYT1, which are among the close regulators of MPF. Nevertheless, the dual specificity cell division cycle 25 phosphatase (CDC25), which is the main activator of MPF, is clearly impacted since its S-nitrosylation annihilates its phosphatase activity ([71, 74]; Gelaude and Bodart: personal observations).

Beyond the cell cycle regulators, S-nitrosylation has been called to play a role in preimplantation embryos and implantation. Microenvironmental presence of NO was reported to contribute to the pathologic effects of endometriosis on the development potential of embryos. In this context, NO effects on embryo survival could either rely upon S-nitrosylation, NO/GC/cGMP or peroxynitrite formation. Lee et al. [50] suggested that the apoptotic effects of excessive NO on embryos were related to S-nitrosylation rather than to any other mechanisms. These effects were closely associated with lipid-rich organelles (mitochondria and endoplasmic reticulum) [50, 75]. Regarding implantation, NO was shown to influence trophoblasts motility [76, 77]. It was further suggested that the effects of NO on trophoblast migration and invasion, which are critical processes for the successful embryonic development, were mediated by nitrosylation of the matrix metalloprotease MMP9 [78]. Indeed, while MMP9 has been reported to be nitrosylated [79], it was colocalized with iNOS and S-nitrosylated proteins at the leading edge in trophoblast [78]. Finally, the trophoblast also appeared to be protected from apoptosis* via* S-nitrosylation of caspase 3 [80].

Thus, S-nitrosylation of proteins might play pivotal roles throughout the early development, modulating cell cycle, trophoblast motility, and embryo survival (Table 1).

### 3.2. S-Sulfhydration as Another Modulator of Enzymatic Activities

The impacts of H_2_S and S-sulfhydration have been addressed and considered to a lesser extent, mainly due to the lack of methodologies [81]. Since the specification of protein S-sulfhydration sites has been enabled, increasing evidence has come to underline the ability of S-sulfhydration to enhance or impair an enzymatic activity. S-sulfhydration was reported to impair the activity of KEAP1 [82], while it increases the activity of K_ATP_ and Ca^2+^ channels, glyceraldehyde 3-phosphate dehydrogenase (GAPDH), nuclear factor *κ*B (NF-*κ*B), and MAPK/ERK kinase 1 (MEK1) [57, 61, 82–84]. In addition to the above-mentioned S-sulfhydrated proteins, S-sulfhydration of cystathionine *β*-synthase (CBS) and cystathionine *γ*-lyase (CSE), H_2_S-releasing enzymes, has been observed [57] and existence of feedback in H_2_S production is supported.

Protein phosphatase serves as points of flexibility and crucial regulation in network signalling. Evidence had raised the fact that it might be particularly subject to S-sulfhydration. Among the phosphatase types involved in early embryogenesis and/or signalling pathways and whose activity might be modulated by S-sulfhydration are phosphatase and tensin homolog (PTEN), protein-tyrosine phosphatase 1B (PTP1B), and aforementioned CDC25. Protein phosphatase PTEN is requested at early steps for proper embryonic development [85]. In the case of PTEN, S-sulfhydration was reported to maintain the activity of the phosphatase [86], by preventing its S-nitrosylation, which would result in protein degradation [87]. PTP1B belongs to the family of ErbB, involved in numerous signalling pathways modulating proliferation, adherence, migration, or survival. PTP1B was shown to be inactivated by S-sulfhydration of cysteine C215, located in its catalytic site [88].

Also, CDC25 might be sulhydrated and inactivated presumably by modification of the cysteine in its active site [89]. There is no direct evidence for CDC25 sulfhydration, but since organosulphur compounds inhibit CDC25A and promote G2/M arrest [90] and CDC25 are targeted by ROS and S-nitrosylation, CDC25 are likely to be S-sulfhydrated [91]. Further studies are obviously needed to gather an exhaustive list of S-sulfhydrated proteins, and one might first focus on proteins, which have been already reported as being S-nitrosylated. MKP1, ERK1, CDK2 and CDK5, CDC25, and MMP9 appear as appealing candidate (Table 1). Indeed, evidences have been raised for cross-talk between S-sulfhydration and S-nitrosylation for many proteins.

### 3.3. A Cross-Talk of S-Sulfhydration and S-Nitrosylation?

Many protein sites have been reported to undergo either S-nitrosylation or S-sulfhydration. As an example, the residue cysteine C150 in GAPDH had been found either S-nitrosylated or S-sulfhydrated [54, 92–94]. Susceptibility for both modifications may strike root in the chemical properties of the involved thiols by S-nitrosylation and S-sulfhydration [81]. If S-sulfhydration and nitrosylation can occur on reactive cysteine residues, they frequently involve the same residue, generally by promoting different and opposing effects. Indeed, S-nitrosylation typically reduces cysteine thiols reactivity while S-sulfhydration increases cysteine thiols reactivity, thereby making them more nucleophilic. For instance, S-sulfhydration and nitrosylation on the same sites have been reported for GAPDH, Parkin, and the p65 subunit of NF-*κ*B (nuclear factor-*κ*B) (Table 1). The increase of GAPDH activity stimulated by S-sulfhydration is antagonized by nitrosylation, which impairs the glycolytic activity of the enzyme [54, 92–94]. Similarly for Parkin, the S-nitrosylation impairs the enzyme activity whereas sulfhydration stimulates it [95, 96].

S-nitrosylation and sulfhydration both regulate the p65 subunit of the antiapoptotic transcription factor NF-*κ*B, which provided quite a school-case for the interplay of S-nitrosylation and sulfhydration [82]. S-sulfhydration of NF-*κ*B has been reported to inhibit apoptosis. Persulfidation of cysteine 38 of p65 unit of NF-*κ*B promotes binding of NF-*κ*B to the coactivator ribosomal S3, thereby increasing its binding to promoters of antiapoptotic genes. Also, cysteine 38 persulfidation might function as the molecular “key” by which hydrogen sulfide prevents NF-*κ*B pathway activation in ox-LDL-induced macrophage inflammation by impairing NF-*κ*B p65 phosphorylation, nuclear translocation, and, therefore, DNA binding activity [97]. One has to note that NF-*κ*B S-sulfhydration may not account for all the protective effects of H_2_S towards inflammation. Subsequent to sulfhydration, nitrosylation of p65 reversed the activation of NF-*κ*B targets [98, 99].

Similarly, actin, whose modifications of properties are requested for the rapid cadence of cytokinesis during early embryogenesis, is nitrosylated or sulfhydrated. While S-sulfhydration of actin resulted in an increase of filament polymerization [57], S-nitrosylated actin exhibited a decrease in polymerization activity and thus an impairment in actin network formation [100, 101]. Actin-binding proteins such as profilin [101] and cofilin [102] are also subject to S-nitrosylation and may contribute through the latter modifications to modulate the remodelling of the actin network. Thus, if we are to compare S-sulfhydration and nitrosylation, we should mainly outline that (1) proteins are rather S-sulfhydrated than S-nitrosylated and (2) nitrosylation is more likely to inhibit and impair protein functions (Table 1).

One may also hypothesize that the sequence of S-nitrosylation and sulfhydration could provide a way for a fine tuning of signalling pathways and cellular functions regulation. Because protein S-nitrosylation can foster intramolecular disulfide bond formation, a protein S-nitrosylation event might promote the formation of a more enduring S-sulfhydration reaction. Moreover, S-sulfhydration of eNOS and its increased activity have been described [103]. Supervision of Ca^2+^ influx and availability of eNOS, Ca^2+^-dependent, is another mechanism of H_2_S-controlled NO creation [104]. Likewise, reverse NO modulation effect on H_2_S releasing is assumed; however, it has not been uncovered so far.

## 4. Perspectives of Gasotransmitters for Assisted Reproductive Technologies

### 4.1. About Recent Reproductive Medicine

With respect to the above-described posttranslational modifications, the causality of some of the phenomena is explained. Assisted reproductive technologies (ART), as a medicinal approach to the solution of human infertility, are a field where the posttranslational modifications and their consequences could be utilized.

Embryos by produced* in vitro* by ART show differences compared to the* in vivo* grown embryos. Routinely used ART techniques, such as* in vitro* fertilization (IVF) or intracytoplasmic sperm injection (ICSI), may affect embryonic development differentially on cellular and molecular levels. Moreover, individual approaches are not equal where a slight delay of early embryonic development of IVF-produced human embryos compared to those fertilized by ICSI has been described [105, 106]. An alteration of embryonic chromatin modifications, including posttranslational modifications of nucleosomal proteins, essential for genome reprogramming and successful development into blastocyst, is a possible explanation of this phenomenon [107–109]. To this respect, gasotransmitter involvement could be justly considered.

Supplementation of culture media with donors of NO or H_2_S improves embryonic development* in vitro* [20, 58, 110]. The necessity of physiological NO production has been demonstrated for* in vitro* fertilization and embryo culture [111–113]. Moreover, NO plays a role in the second meiotic block release and oocyte activation [42, 43, 114]. The suppression of H_2_S physiological production even leads to oocyte maturation failure [59]. Therefore, precise supplementation of NO and H_2_S allows optimizing fertilization conditions [112] as well as relieving embryonic development defects. However, the molecular mechanisms are not known and target system is questionable.

### 4.2. Epigenetic Dimension of Gasotransmitters

S-nitrosylation, one of the above-mentioned NO-derived protein posttranslational modifications, affects direct chromatin modification, namely, tyrosine nitration of nucleosomal core histones [115]. Histone modification* via* NO is supposed to be decisive for gene activity [116]; however, final effect of histone nitration remains unclear [117]. In addition to S-nitrosylation, core histone is affected by acetylation and methylation [118, 119] and some evidence (mentioned below) indicates that NO and/or H_2_S could be indirectly required in upstream signalling of these modifications. Accordingly, influencing histone modifying upstream enzymes is another NO/H_2_S-modulated chromatin gene activity. Hereby, NO interacts with histone deacetylases (HDACs) when their activity is inhibited by NO in neurons [120]. On the other hand, NO is capable of activating Sirtuin 1 (SIRT1), one of NAD^+^-dependent HDACs [121]. Similarly, H_2_S has been described as a potent activator of SIRT1 [122]. Presumably, S-nitrosylation and S-sulfhydration are responsible for NO and H_2_S effect, respectively. On the other hand, NO/H_2_S-derived modifications do not seem to be strictly upstream, because H_2_S releasing stimulated by resveratrol, a strong activator of SIRT1, has been recently observed [123].

The above-mentioned SIRT1 is responsible for modifications of both histone and nonhistone targets [124–126] and through modulation of its activity it brings a broad spectrum of S-nitrosylation and sulfhydration effect. In addition to histone deacetylation, complex SIRT1 signalling leads to histone methylation and thus chromatin stabilization, which is, however, accompanied by gene silencing (summarized in [127]). Apparently, gasotransmitters are involved in wide epigenetic regulations, affecting gene expression without changes in gene sequences themselves. Some evidence indicates targeted chromatin modulation and transcriptional activation of certain genes [128, 129], due to a molecular mechanism which is yet unknown.

### 4.3. Delicacy of Gasotransmitter Involvement in Epigenetic Regulation

In contrast to the lifespan beneficial genome stability, embryonic genome reprogramming requires transcriptional activity, nevertheless, followed by DNA damage-prone euchromatin creation, marked by histone acetylation [132–135]. Therefore the equilibrium between chromatin stability and transcriptional activity is obviously the compromise for successful embryonic development. The dual effect of NO and H_2_S on HDACs and NAD^+^-dependent HDACs [136, 137] and the delicate balance between them, obvious in somatic cells [138], could be the key to embryonic genome activation and impeccable further embryogenesis.

In accordance with presumption of H_2_S-epigenetically affected embryogenesis, cell cycle and proliferation are affected by H_2_S as well [139, 140]. The involvement of H_2_S in regulation of specific promoters has been described in vascular smooth muscle cells [141]. Interestingly, ten-eleven translocation (Tet) proteins, factors playing a role in epigenetics of early embryo [142], are included in described H_2_S-modulated genes [143]. Although there are evidences of H_2_S-derived epigenetic regulation of cell cycle, the characterization of H_2_S-caused chromatin modifications remains clean.

In general, presence of NO and H_2_S has been reported in mammalian oocytes and embryos as well [32, 59, 144] and their cross-talk due to S-nitrosylation and sulfhydration was reported, where their necessity is assumed. Meanwhile, there is poor knowledge of all gasotransmitters' potentiality, for example, (a) direct H_2_S-derived S-sulfhydration of core histones, (b) NO/H_2_S/SIRT1 axis, leading to chromatin equilibrium between an adequate transcriptional gene activity and genome stability, and (c) absent knowledge of CO involvement in epigenetics-driven embryogenesis. Regarding CO, its molecular action remains fully unidentified and CO-derived modifications have not yet been completely explained.

Obviously, understanding the molecular mechanism of NO/H_2_S interaction and HDACs-modified embryonic chromatin offers a possibility for improvement of* in vitro* embryo production* via* a gasotransmitter tool. A complete understanding of the cross-talk between all gasotransmitters, including CO, is necessary and a holistic approach should be emphasized.

## 5. Conclusion

This review summarizes the recent knowledge of gasotransmitters' action in maturing oocytes and early embryonic development, in various animal species, including sea urchin,* Xenopus,* and mammalian models. Current observations point out the necessity of NO and H_2_S in these processes; however, the role of CO remains unexplained.

Based on our best knowledge, the observations, performed on amphibian and mammalian female reproduction, enlightened various species-specific biological action of both NO and H_2_S. Nevertheless, the gasotransmitter-derived posttranslational modifications are shared throughout the studied animal models. Both S-nitrosylation and S-sulfhydration may be required for adequate protein activities/functions and therefore, patterns of posttranslational modifications create NO- and H_2_S-modulated proteome in oocytes and embryos. Importantly, most of gasotransmitter-modified proteins may not have been yet described. Although the understanding is limited, S-nitrosylation and sulfhydration seem to be equal to other posttranslational modifications' impact. In contrast to the wide spectrum of kinases mediating phosphorylation and regulation of various proteins, NO and H_2_S decide on the activity of a comparable spread of proteins. However, other alternative molecular mechanisms could be considered, often epoch-making, such as possible ROS-generating H_2_S due to RSS creation [62, 63].

In addition to above-mentioned absence of insight, CO, the third known gasotransmitter, is still unexplored and its molecular involvement in gametogenesis and embryogenesis waits for verification. The principle of CO molecular action is unknown and NO and H_2_S like posttranslational modifications can be presumed. The evolutionary permanence of CO biological effect is questionable, with respect to the existing recognition traits of NO and H_2_S. In addition to the single CO action, the interaction of all gasotransmitters offers infinite consequences resulting in various effects in gametes and embryos. Three gasotransmitters have been described so far and some other small molecules, such as sulphur dioxide [145, 146] or hydrogen [147, 148], exhibit possible gasotransmitter features as well.

Obviously, the understanding and further study of gasotransmitters are necessary for the advancement of human ART. The* in vitro* technologies are based on a simulation of* in vivo* conditions, still lacking undefined factors. Gasotransmitters are among the essential molecules, missing in* in vitro* protocols where their failure is appreciable. However, their volatility makes them difficult to supplement into culture media and the development of an applicable gasotransmitter treatment is subject to research. A serious consideration of gasotransmitters as signal molecules, respecting their evolutionary consequences, represents an expectation for current therapy of human reproduction.

## Figures and Tables

**Table 1:** Examples of S-nitrosylated and/or S-sulfhydrated proteins.

Protein	Sulfhydration site	Sulfhydration effect on function	Nitrosylation site	Nitrosylation effect on function	References
MKP1	n.d.	n.d.	C258	Stability of protein	Guan et al., 2012 [130]

ERK1	n.d.	n.d.	C183 (potential)	Prevention of phosphorylation	Feng et al., 2013 [131]

CDK2	n.d.	n.d.	n.d.	Increase of kinase activity	Kumar et al., 2010 [72]

CDK5	n.d.	n.d.	n.d.	n.d.	Foster et al., 2009 [71]

CDC25	n.d.	n.d.	n.d.	Loss of phosphatase activity	Foster et al., 2009 [71]; Majumdar et al., 2012 [74]

MMP9	n.d.	n.d.	n.d.	Increase of activity	Harris et al., 2008 [78]

PTP1B	C215	Reduction of phosphatase activity	n.d.	n.d.	Krishnan et al., 2011 [88]

PTEN	C71, C124	Maintenance of enzyme activity and prevention of further oxidation by NO	C83	Promotion of survival signal and protein degradation	Kwak et al., 2010 [87]; Ohno et al., 2015 [86]

Actin	n.d.	Increase of polymerization activity	Cys 374	Decrease in polymerization activity and network formation	Dalle-Donne et al., 2000 [100]; Mustafa et al. 2009 [57]; Thom et al., 2008 [101]

MEK1	C341	Facilitation of Parp activation	n.d.	Loss of kinase activity	Ben-Lulu et al., 2014 [73]; Zhao et al., 2014 [61]

Parkin	n.d.	Increase of activity	n.d.	Decrease of activity	Chung et al., 2004 [95]; Vandiver et al., 2013 [96]

GAPDH	C150	Increase of the activity sevenfold	C150	Inhibition of glycolytic activity	Greco et al., 2006 [93]; Hao et al., 2006 [94]; Hara et al., 2005 [92]; Mustafa et al., 2009 [57]

## Abbreviations

APC/C: Anaphase promoting factor/cyclosome
ART: Assisted reproductive technologies
CaMKII: Calmodulin-dependent protein kinase II
CBS: Cystathionine *β*-synthase
CDC25: Cell division cycle 25 phosphatase
CDKs: Cyclin-dependent kinases
CSE: Cystathionine *γ*-lyase
GAPDH: Glyceraldehyde 3-phosphate dehydrogenase
HDACs: Histone deacetylases
ICSI: Intracytoplasmic sperm injection
IVF: * In vitro* fertilization
MAPK: Mitogen-activated protein kinase
MEK1: MAPK/ERK kinase 1
MPF: M-phase/maturation promoting factor
3-MPST: 3-Mercaptopyruvate sulphurtransferase
NF-*κ*B: Nuclear factor *κ*B
eNOS: Endothelial nitric oxide (NO) synthase
nNOS: Neuronal nitric oxide (NO) synthase
iNOS: Inducible nitric oxide (NO) synthase
PKA: Protein kinase A
PKG: Protein kinase G
PLKs: Polo-like kinases
PTEN: Phosphatase and tensin homolog
PTP1B: Protein-tyrosine phosphatase 1B
ROS: Reactive oxygen species
RSS: Reactive sulfide species
sGC: Soluble guanylate cyclase
SIRT1: Sirtuin 1, SirT1, NAD^+^-dependent HDAC 1.
